# DNA methylation of FKBP5 and response to exposure‐based psychological therapy

**DOI:** 10.1002/ajmg.b.32650

**Published:** 2018-10-18

**Authors:** Susanna Roberts, Robert Keers, Gerome Breen, Jonathan R. I. Coleman, Peter Jöhren, Agnieszka Kepa, Kathryn J. Lester, Jürgen Margraf, Silvia Scheider, Tobias Teismann, André Wannemüller, Thalia C. Eley, Chloe C. Y. Wong

**Affiliations:** ^1^ King's College London, Social, Genetic and Developmental Psychiatry Centre Institute of Psychiatry, Psychology and Neuroscience London United Kingdom; ^2^ Queen Mary University of London School of Biological and Chemical Sciences London United Kingdom; ^3^ National Institute for Health Research Biomedical Research Centre South London and Maudsley National Health Service Trust United Kingdom; ^4^ Dental Clinic Bochum Bochum Germany; ^5^ University of Sussex Department of Psychology Brighton United Kingdom; ^6^ Ruhr‐Universität Bochum Mental Health Research and Treatment Center Bochum Germany

**Keywords:** anxiety, cognitive behavioral therapy, epigenetics, treatment response

## Abstract

Differential DNA methylation of the hypothalamic‐pituitary‐adrenal axis related gene *FKBP5* has recently been shown to be associated with varying response to environmental influences and may play a role in how well people respond to psychological treatments. Participants (*n* = 111) received exposure‐based cognitive behavioural therapy (CBT) for agoraphobia with or without panic disorder, or specific phobias. Percentage DNA methylation levels were measured for the promoter region and intron 7 of *FKBP5*. The association between percentage reduction in clinical severity and change in DNA methylation was tested using linear mixed models. The effect of genotype (rs1360780) was tested by the inclusion of an interaction term. The association between change in DNA methylation and *FKBP5* expression was examined. Change in percentage DNA methylation at one CpG site of intron 7 was associated with percentage reduction in severity (β = −4.26, *p* = 3.90 × 10^−4^), where a decrease in DNA methylation was associated with greater response to therapy. An interaction was detected between rs1360780 and changes in DNA methylation in the promoter region of *FKBP5* on treatment outcome (*p* = .045) but did not survive correction for multiple testing. Changes in DNA methylation were not associated with *FKBP5* expression. Decreasing DNA methylation at one CpG site of intron 7 of *FKBP5* was strongly associated with decreasing anxiety severity following exposure‐based CBT. In addition, there was suggestive evidence that allele‐specific methylation at the promoter region may also be associated with treatment response. The results of this study add to the growing literature demonstrating the role of biological processes such as DNA methylation in response to environmental influences.

## 1 INTRODUCTION

Research examining genetic and biological factors involved in response to psychological therapies has gained momentum in recent years. A number of studies have identified candidate gene predictors of therapy response, although results are inconsistent and have been of small effect (Lester & Eley, 2013). Identification of the potential biological mechanisms of response has led researchers to explore the epigenetic process of DNA methylation. Early studies in this area have demonstrated that changes in DNA methylation may underlie response to psychological therapies (e.g., Perroud et al., 2013; Roberts et al., 2014; Ziegler et al., 2016). Of particular relevance for the current study, changes in DNA methylation at the promoter region of *FKBP5* have also been implicated in response to exposure therapy in veterans with post‐traumatic stress disorder (PTSD) (Yehuda et al., 2013), with decreases in region‐specific DNA methylation across the course of therapy associated with better response and increases with poorer response. In addition, we previously reported allele‐specific changes in *FKBP5* promoter methylation were associated with response to Cognitive Behavioural Therapy (CBT) in children with anxiety disorders (Roberts et al., 2015). Specifically, a better response to therapy was associated with a decrease in DNA methylation, but only in individuals with *FKBP5* risk genotypes.

*FKBP5* plays a critical role in reactivity to stress through negative regulation of the hypothalamus‐pituitary‐adrenal (HPA) axis. Stress exposure rapidly stimulates glucocorticoid secretion and termination of this response involves binding of glucocorticoids such as cortisol at the glucocorticoid receptor. *FKBP5* acts as a functional negative regulator of glucocorticoid receptor sensitivity by reducing binding affinity and restricting nuclear translocation (Denny, Valentine, Reynolds, Smith, & Scammell, 2000; Wochnik et al., 2005). An ultra‐short negative feedback loop is created by the regulation of *FKBP5* mRNA expression by glucocorticoids via glucocorticoid response elements. Dysregulation of this process has been widely implicated in stress related disorders such as anxiety disorders (Binder, 2009; Holsboer, 2000; Pariante & Miller, 2001).

Studies of *FKBP5* show support for an interaction between negative/stressful environments and genotype (including the SNP rs1360780) predicting adverse mental health outcomes, such as PTSD, depression and suicidality (Appel et al., 2011; Binder et al., 2008; Koenen et al., 2005; Roy, Gorodetsky, Yuan, Goldman, & Enoch, 2010; Zimmermann et al., 2011). Further research investigating the potential mechanism underlying this gene‐by‐environment interaction has identified allele‐specific DNA methylation patterns following childhood trauma (Klengel et al., 2013). It is thought that this association is facilitated by genotype‐dependent structural differences, leading to functional alterations in the responsiveness of *FKBP5* to glucocorticoid receptor activation. The involvement of *FKBP5* methylation in response to both positive environments (e.g., psychological therapy, Roberts et al., 2015; Yehuda et al., 2013) and negative environments (e.g., childhood trauma, Klengel et al., 2013) suggests that epigenetic variation in this gene may represent a marker of differential susceptibility – that is, sensitivity to the environment “for better or for worse” (Belsky et al., 2009).

In this study, we examined the association between changes in DNA methylation in two regions of *FKBP5* and therapy outcome at posttreatment and follow‐up in a sample of adults (*n* = 111) with fear‐related anxiety diagnoses receiving exposure‐based CBT. In addition, we utilized genetic data (*FKBP5* SNP rs1360780) to test for allele‐specific effects of DNA methylation changes. This study explores response to a psychological therapy across the whole treatment period, and builds on previous research demonstrating that epigenetic changes in *FKBP5* may play a role in treatment response (Roberts et al., 2015; Yehuda et al., 2013). It is the first study that examines DNA methylation changes at intron 7 of *FKBP5* with respect to psychological therapy response, and the first to combine genetic, epigenetic and gene expression data.

## 2 METHODS

### 2.1 Sample & treatment

Participants (*n* = 111) were recruited at Mental Health Research and Treatment Center, Ruhr‐Universität Bochum, Germany (*n* = 61, 55.0%) or the Dental Clinic Bochum, Germany (*n* = 50, 45.0%). Age at baseline ranged from 19 to 68 years (mean = 39.9 years), and 67.3% of the sample were female. All participants were treated for agoraphobia with or without panic disorder (*n* = 34, 30.6%) or specific phobia (*n* = 77, 69.4%; including dental fear – *n* = 50, 45.0% of total). The full study sample reported here includes participants with these fear‐related anxiety disorders as exposure is a key element of treatment for such diagnoses. Diagnoses were made according to DSM‐IV criteria by trained clinicians using the Diagnostisches Interview bei Psychischen Störungen (DIPS) and mini‐DIPS (Margraf, 1994; Margraf, Schneider, & Ehlers, 2013; Schneider & Margraf, 2011). At pretreatment, 33.3% (*n* = 37) were smokers, 6.3% (*n* = 7) were using a form of psychoactive medication, and 33.3% (*n* = 37) took other regular medications.

All participants completed one of four exposure therapy or exposure‐based CBT treatment programs as detailed below and shown in Supporting Information Figure S1. Exclusion criteria included psychotic symptoms and presence of severe learning difficulties. Treatment was administered at the Mental Health Research and Treatment Centre in three groups. All participants received five preliminary sessions covering diagnosis and psychoeducation before starting therapy. Participants with a primary diagnosis of panic disorder were randomized either to exposure‐based CBT (*n* = 17) or to an exposure‐alone condition without any element of cognitive restructuring (*n* = 17) [Clinical Trials: NCT01680327]. Participants with specific phobia (not primarily associated with dental fear, *n* = 27) were treated in a program of up to 25 sessions of *in vivo* exposure. Participants in these groups were excluded if they were using anxiolytic medication. Individuals with high levels of dental fear (*n* = 50) were treated in a dental anxiety‐specific program at the Dental Clinic Bochum. Treatment was given in five sessions, including an initial diagnostic and psychoeducation session, and a session developing relaxation techniques and focusing on helpful thoughts. These coping strategies were then encouraged in three sessions of consisting of exposure scenarios such as video exposure, noise exposure and *in sensu* exposure (virtual reality or visualization). Concurrent psychoactive medication was not an exclusion criterion.

### 2.2 Outcome measures

Treatment response was defined as change in clinician rated severity of the treated diagnosis, as determined using the Clinical Global Impression – Severity (CGI‐S) scale. The CGI‐S consists of a scale of 1–7, with a score of 1 indicating that the patient is healthy, and 7 indicating that that the patient is extremely ill. Scores were transposed to a scale of 0–6, and percentage improvement from pre to post‐treatment and pretreatment to follow‐up was calculated for all participants. The mean number of days from pre‐ to post‐treatment was 229.9 (SD: 201.8; range: 19–736). The mean interval from post‐treatment to follow‐up was 219.1 (SD: 74.1; range: 87–640).

### 2.3 Sample collection and extraction

Whole blood samples were drawn using EDTA tubes at pretreatment, posttreatment, and follow‐up (usually ∼6 months following the conclusion of treatment; mean 219 days). DNA was extracted using the FlexiGene DNA kit (Qiagen, Hilden, Germany). Extracted samples were quantified using spectrophotometry (Nanodrop 1000; Thermo Fisher Scientific, Waltham, MA).

### 2.4 Genotyping

#### 2.4.1 FKBP5

The *FKBP5* SNP rs1360780 was genotyped by LGC Genomics (Hoddesdon, UK) using KASP technology with validated arrays. Genotypic distribution of the samples conformed to Hardy–Weinberg proportions (CC = 50.5%, CT = 40.5%, TT = 9.0%; χ^2^_1_ = .049, *p* = .820). Genotype was coded to reflect a dominant model (CC vs. CT/TT; no risk alleles vs. 1+ risk alleles) as in previously reported studies (Binder et al., 2008; Klengel et al., 2013).

### 2.5 DNA methylation

#### 2.5.1 Measurement

Extracted DNA (500 ng) was treated with sodium bisulfite using the EZ‐96 methylation kit (Zymo Research, Irvine, CA). polymerase chain reaction (PCR) amplification of bisulfite‐treated DNA was performed using Sequenom MassCLEAVE tagged primers (designed using the Sequenom EpiDesigner software, Supporting Information Table S1), and Qiagen HotStarTaq DNA polymerase, with 35 cycles and an annealing temperature of 63°C. Two regions of interest were assayed based on previous literatures; first, the promoter region (chr6:35,695,823‐35,696,542 UCSC NCBI37/h19) as examined in Roberts et al. (2015); second, intron 7 (chr6:35,558,191‐35,558,904 UCSC NCBI37/h19) which corresponds to the region defined in Klengel et al., (2013). Further information regarding the genomic location of these amplicons is provided in the supporting information to this article (Supporting Information Figures S2 and S3). Percentage DNA methylation was quantitatively measured using the Sequenom EpiTyper system (Sequenom, San Diego, CA). Change in percentage DNA methylation was calculated for the pre‐ to post‐treatment and pretreatment to follow‐up periods (later time‐point minus earlier time‐point).

#### 2.5.2 Quality‐control

To minimize batch effects, all time‐points for each participant were included in the same plate for bisulfite conversion, PCR amplification and Sequenom array. Bisulfite conversion and PCR amplification were both conducted in duplicate and the products pooled at each stage. Fully methylated and fully unmethylated samples were included as technical controls. All samples were processed blind to sample identification. Data generated from the EpiTYPER software were treated with stringent quality‐control analysis. Probes detecting an average percentage DNA methylation of <5% were excluded from analyses. Further probes with >15% missingness overall were also excluded. Finally, only participants with DNA methylation values at pretreatment and posttreatment, follow‐up, or both were included in analyses (*n* = 110 included in at least one analysis, numbers vary by analysis). After stringent quality‐control, quantitative DNA methylation data from intron 7 (covering 5 CpG sites) and the promoter region (covering 2 CpG sites) were used for downstream analysis. Details of the location of these probes can be found in the Supporting Information Figures S2 and S3.

### 2.6 Gene expression

Gene expression levels for *FKBP5* at all time‐points were taken from previously quality‐control processed data from whole blood samples quantified using the Illumina HT‐12 v4 BeadChip array (*n* = 102). Full details of data preparation and quality‐control procedures are reported elsewhere (Roberts et al., 2017). Briefly, this involved background correction and filtering based on probe intensity and detection rates. Probes were then transformed and normalized, and outliers identified and removed.

### 2.7 Ethics statement

This study was conducted in accordance with the principles outlined by the Declaration of Helsinki. Site‐specific trials and the collection of samples were approved by local Human Ethics and Biosafety Committees, and all participants provided informed consent. The receipt, storage and analysis of samples were approved by the London‐Bentham NRES Committee and the King's College London Psychiatry, Nursing and Midwifery Research Ethics Sub‐Committee.

## 3 ANALYSES

### 3.1 Confounding factors

Baseline severity, age, BMI, gender, smoking status, psychoactive medication status, and other medication status were tested for association with percentage reduction in clinical severity and change in DNA methylation between the time‐points tested (Tables 1, 2, 3). In order to control for potential population stratification, the first two principal components (“PC1” and “PC2”) from genome‐wide genotyping data (details reported elsewhere; Coleman et al., 2016) were also tested for association with treatment outcome. Variables displaying a significant association with either treatment outcome or change in DNA methylation (*p* < .05) were included as covariates in the relevant analyses (Tables 1, 2, 3).

**Table 1:** Potential confounding variables associated with treatment outcome

	Percentage change in CGI‐S severity			
	Post‐treatment		Follow‐up	
Clinical factor	*R*	*p*	*r*	*p*
Baseline severity	−.042	.662	.169	.089
Age	−.070	.471	.041	.680
BMI	.059	.543	−.038	.703
	*t (df)*	*p*	*t (df)*	*p*
Gender	−.559 (107)	.577	.089 (100)	.929
Smoking status	.212 (107)	.832	−.010 (100)	.992
Psychoactive medication	4.418 (107)	<.001*	3.150 (100)	<.001*
Other medications	1.308 (107)	.194	1.17 (100)	.245
**Population stratification**	***r***	***p***	***r***	***p***
PC1	−.163	.097	−.024	.817
PC2	−.038	.700	.051	.615

N.B *Significant and included as a covariate in analyses.

**Table 2:** Potential confounding variables associated with change in DNA methylation from pre‐ to post‐treatment

	Intron 7: change pre‐ to post‐treatment												Promoter: change pre‐ to post‐treatment			
	CpG 1		CpG 2		CpG 3		CpG 4		CpG 5		Bin 2		CpG 1		CpG 2	
Clinical factor	*r*	*p*	*r*	*p*	*r*	*p*	*r*	*p*	*r*	*p*	*r*	*p*	*r*	*p*	*r*	*p*
Baseline severity	−.038	.728	−.034	.754	−.018	.86	−.151	.144	−.043	.691	−.123	.238	−.072	.484	−.03	.777
Age	−.022	.843	−.026	.806	.097	.336	−.099	.341	−.067	.532	.039	.713	−.01	.957	.014	.894
BMI	−.098	.369	−.087	.42	.123	.222	.085	.415	.068	.526	.1	.339	.034	.746	−.008	.942
	*t (df)*	*p*	*t (df)*	*p*	*t (df)*	*p*	*t (df)*	*p*	*t (df)*	*p*	*t (df)*	*p*	*t (df)*	*p*	*t (df)*	*p*
Gender	−.497(84)	.620	1.00(87)	.320	.546(98)	.586	−1.727(93)	.088	.839(87)	.404	.454(92)	.651	.286(94)	.775	.794(92)	.429
Smoking status	−1.639(84)	.105	.347(87)	.730	.095(98)	.925	−.853(93)	.396	−.584(87)	.561	−.190(92)	.850	−.674(94)	.502	−.772(92)	.442
Psychoactive medication	−0.415(84)	0.679	−0.641(87)	0.523	−0.337(98)	0.737	−0.466(93)	0.642	−0.409(87)	0.684	−0.574(92)	0.567	−0.510(94)	0.612	−0.392(92)	0.696
Other medications	−.750(84)	.455	−.188(87)	.851	−.720(98)	.474	.346(93)	.730	−.259(87)	.796	−.155(92)	.878	−.817(94)	.416	−.751(92)	.455
**Population Stratification**	***r***	***p***	***r***	***p***	***r***	***p***	***r***	***p***	***r***	***p***	***r***	***p***	***r***	***p***	***r***	***p***
PC1	−.022	.842	.016	.885	−.183	.073	−.171	.103	.056	.611	−.277	.008*	−.161	.124	−.116	.276
PC2	.053	.636	.033	.763	.003	.981	.131	.214	.127	.244	.061	.568	−.005	.964	−.062	.562

N.B *Significant and included as a covariate in analyses.

**Table 3:** Potential confounding variables associated with change in DNA methylation from pre‐treatment to follow‐up

	Intron 7: change pre‐treatment to follow‐up												Promoter: change pre‐treatment to follow‐up			
	CpG 1		CpG 2		CpG 3		CpG 4		CpG 5		Bin 2		CpG 1		CpG 2	
Clinical factor	*r*	*p*	*r*	*p*	*r*	*p*	*r*	*p*	*r*	*p*	*r*	*p*	*r*	*p*	*r*	*p*
Baseline severity	−.152	.167	−.005	.967	.053	.603	−.083	.429	.022	.841	−.034	.753	−.14	.173	−.093	.37
Age	.063	.57	−.028	.803	.261	.010*	.255	.014*	.143	.184	.235	.025*	.062	.548	.043	.677
BMI	−.089	.42	−.098	.376	.166	.102	.218	.036*	.03	.78	.179	.091	.039	.708	.065	.53
	*t (df)*	*p*	*t (df)*	*p*	*t (df)*	*p*	*t (df)*	*p*	*t (df)*	*p*	*t (df)*	*p*	*t (df)*	*p*	*t (df)*	*p*
Gender	−.528(82)	.599	.650(82)	.517	.443(96)	.659	.132(91)	.896	1.230(86)	.222	.481(89)	.632	.795(94)	.429	1.465(93)	.146
Smoking status	−.209(82)	.835	.745(82)	.459	−.147(96)	.884	−.621(91)	.537	−.111(86)	.912	−.342(89)	.734	1.909(94)	.059	2.002(93)	.048*
Psychoactive medication	−.264(82)	.793	−.233(82)	.816	−.492(96)	.624	−.691(91)	.491	.135(86)	.893	−.735(89)	.465	−.531(94)	.597	−.317(93)	.752
Other medications	.442(82)	.660	.276(82)	.783	−2.029(96)	.045*	−1.880(91)	.063	.368(86)	.714	−2.101(89)	.039*	−1.473(94)	.144	−.765(93)	.446
**Population Stratification**	***r***	***p***	***r***	***p***	***r***	***p***	***r***	***p***	***r***	***p***	***r***	***p***	***r***	***p***	***r***	***p***
PC1	.041	.719	.083	.459	−.138	.182	−.187	.077	.047	.673	−.216	.043*	−.159	.13	−.114	.284
PC2	−.092	.416	−.041	.718	−.019	.855	−.207	.05	.088	.422	−.14	.193	−.059	.575	−.08	.45

*Significant and included as a covariate in analyses.

Age was associated with change in DNA methylation from pretreatment to follow‐up for CpG 3, CpG 4, and Bin 2 of Intron 7. Being on other medications was associated with change in DNA methylation from pre‐treatment to follow‐up for CpG 3, and Bin 2 of Intron 7. Smoking was associated with change in DNA methylation from pretreatment to follow‐up for CpG 2 of the promoter region.

### 3.2 DNA methylation, genotype and outcome

In our primary analyses we tested the association between percentage improvement in anxiety disorder severity and contemporaneous change in DNA methylation using linear mixed models. We fitted separate models focusing on change from baseline to the post‐treatment assessment and baseline to follow‐up time‐points, respectively. In addition, to replicate a similar design to our previously reported studies, we also examined the association between change in DNA methylation at *post‐treatment* and subsequent treatment outcome at *follow‐up*.

All individuals with available data were included in each model to maximize sample size. To account for differences between treatment conditions and potential underlying differences in diagnosis groups, treatment group was included as a higher order random effect (four groups, as demonstrated in Supporting Information Figure S1). Fixed effects of time (days from baseline to post, and days from baseline to follow‐up) and number of sessions were included as covariates, as well as variables significantly associated with treatment outcome.

Initially, the association between changes in DNA methylation and treatment outcome were tested (results reported in the Supporting Information Table S2). Allele‐specific effects of these changes were subsequently investigated by including a DNA methylation change by rs1360780 genotype interaction term to the above models. Separate models were run for each CpG probe. Given the previously reported allele‐specific methylation of a group of CpG sites in intron 7 (“bin 2” in Klengel et al., 2013), we also combined CpG probes to give an equivalent group (in this study, data was available for two of the three CpG sites reported previously).

### 3.3 DNA methylation and gene expression

Pairwise correlations were used to examine whether DNA methylation changes were associated with changes in *FKBP5* expression.

### 3.4 Multiple testing correction

Results were considered nominally significant at *p* < .05. A revised significance threshold was estimated to account for the multiple CpG probes being tested in this study. The number of independent variables for DNA methylation changes measured at both post‐treatment and at follow‐up (combined) was calculated using Matrix Spectral Decomposition (MatSpD) to assess the correlations between CpG probes (Nyholt, 2004). The effective number of independent tests was estimated at 14.95, when taking shared variance into account. To account for the additional effect of genotype tested in each interaction analyses, this number was doubled to give 29.9 independent tests, corresponding to a revised significance threshold of *p* < .0016.

## 4 RESULTS

### 4.1 Sample characteristics

From pre‐ to post‐treatment, there was a mean reduction in severity of 69.5% (range: from 100% to −33%). At follow‐up, there was a mean reduction in severity from baseline of 57.1% (range: from 100% to −100%). Being on anxiety medication at pretreatment was associated with treatment outcome (Table 1) and was included as a covariate in all models (medication = 1, no medication = 0). Principal component 1 (PC1) was associated with change in DNA methylation for both time‐points in Bin 2 (Table 2). Other potential confounders, including age, medication and smoking histories, were reported to be associated with change in DNA methylation from pretreatment to follow‐up at particular CpG sites (as detailed in Table 3). These covariates were all included in the relevant analyses.

### 4.2 *FKBP5* DNA methylation and outcome

#### 4.2.1 Intron 7

**Figure 1:**
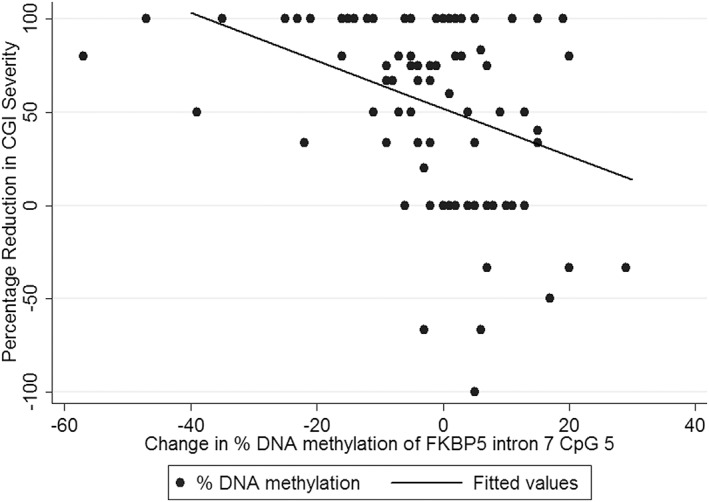
Change in DNA methylation from pre‐ to posttreatment at CpG 5 of intron 7 and percentage reduction in CGI severity at follow‐up. Percentage reduction in CGI severity at follow‐up was significantly associated with change in % DNA methylation from pre‐ to post‐treatment (β = −4.26, *p* = 3.90E‐04)

A significant association with outcome at follow‐up was found for change in DNA methylation during active therapy (from pre‐ to post‐treatment) at CpG 5 of intron 7 (β = −2.51, *p* = 3.90 × 10^−4^; Figure 1, test statistics in Supporting Information Table S2), which remained significant after correcting for multiple testing. At this CpG site, those with a reduction in percentage DNA methylation showed a greater response to treatment (a higher percentage improvement in CGI severity), while individuals with an increase in DNA methylation showed a poorer treatment outcome. Interestingly, change in DNA methylation at this CpG site across the full course of treatment (pretreatment to follow‐up) was also nominally associated with outcome at follow‐up, but this effect did not reach the revised level of significance (β = −1.65, *p* = .024, Supporting Information Table S2).

#### 4.2.2 Promoter

A nominally significant association between outcome at post‐treatment and change in DNA methylation from pre‐ to post‐treatment was found for CpG 1 of the promoter region (post‐treatment: β = 1.74,*p* = .002, Supporting Information Table S2), although this significant effect was not seen for outcome at follow‐up.

### 4.3 DNA methylation, genotype and outcome

**Figure 2:**
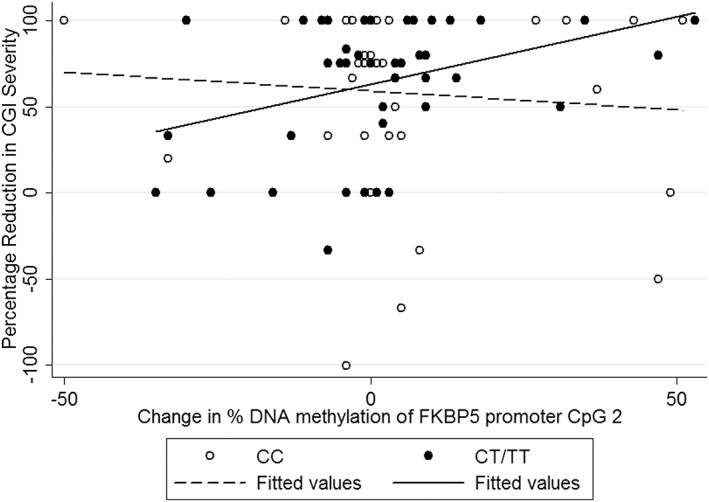
Change in DNA methylation at CpG 2 of the promoter region, rs1360780 genotype and percentage reduction in CGI severity. Percentage reduction in CGI severity from pretreatment to follow‐up and change in % DNA methylation from pre to post‐treatment in CC and CT/TT genotype individuals. Those with the risk genotype (CT/TT) showed a nominally significant association between DNA methylation changes and outcome (β = 1.69, *p* = .007), while no effect was detected in CC carriers (β = −0.36, *p* = .641)

Finally, in order to explore allele‐specific effects of DNA methylation, we tested interactions between rs1360780 and changes in each CpG site on treatment outcomes (see Table 4). We found no evidence for allele‐specific effects of DNA methylation changes in any of the individual CpG sites tested in intron 7 or the previously reported combination of sites (“bin 2”). However, interestingly, a nominally significant interaction between rs1360780 genotype and change in DNA methylation at CpG 2 of the promoter region was detected (β = 2.03, *p* = .044). Post‐hoc exploration of this effect revealed that in individuals with the “risk” genotype (CT/TT), a decrease in percentage DNA methylation was associated with a poor response to treatment, while an increase was associated with a better response (β = 1.73, *p* = .007; Figure 2). In contrast, there was no association between DNA methylation change and treatment outcome for CC genotype individuals (β = −0.36, *p* = .640).

**Table 4:** DNA methylation, rs1360780 and percentage reduction in CGI severity

		Post‐treatment			Follow‐up			Pre‐Post‐treatment, outcome at follow‐up		
Region		β	CI (95%)	*p*	β	CI (95%)	*p*	β	CI (95%)	*p*
Intron 7	CpG 1	1.44	−1.40–4.27	.322	−0.36	−3.12–2.40	.798	−0.28	−3.71–3.15	0.875
	rs1360780	−0.04	−0.45–0.37	.849	0.11	−0.35–0.58	.638	0.17	−0.29–0.62	0.476
	Interaction	−1.09	−5.22–3.04	.606	−0.17	−4.42–4.08	.937	1.72	−2.92–6.36	0.467
	CpG 2	0.6	−1.35–2.55	.546	0.79	−1.47–3.06	.493	1.95	−0.07–3.96	0.058
	rs1360780	−0.06	−0.47–0.34	.764	0.04	−0.42–0.51	.851	0.03	−0.40–0.46	0.877
	Interaction	−0.29	−3.46–2.89	.860	−0.65	−4.37–3.08	.734	−2.36	−5.62–0.89	0.155
	CpG 3	−0.19	−1.10–0.72	.685	−0.93	−1.85–0.01	**.049**	−0.57	−1.55–0.42	0.258
	rs1360780	−0.17	−0.54–0.21	.382	−0.06	−0.46–0.35	.773	−0.11	−0.50–0.28	0.576
	Interaction	−0.45	−2.09–1.18	.586	0.015	−1.50–1.53	.984	−0.77	−2.36–0.82	0.342
	CpG 4	1.57	−1.09–4.24	.248	−1.06	−2.59–0.47	.176	1.29	−1.93–4.51	0.431
	rs1360780	−0.13	−0.51–0.25	.503	0.09	−0.35–0.53	.696	−0.06	−0.49–0.37	0.793
	Interaction	−4.27	−8.87–0.33	.069	1.56	−1.12–4.25	.254	−0.40	−5.55–4.75	0.878
	CpG 5	−0.98	−3.27–1.30	.400	−1.93	−3.76–0.09	**.040**	−4.26	−6.62–1.90	**4.10E‐04**
	rs1360780	0.03	−0.37–0.43	.876	0.03	−0.40–0.45	.902	0.2	−0.20–0.60	0.333
	Interaction	0.06	−2.90–3.02	.967	0.67	−2.14–3.48	.641	2.73	−0.33–5.80	0.080
	Bin 2	1.02	−1.03–3.08	.725	−1.34	−2.92–0.24	.098	−0.71	−3.14–1.72	0.566
	rs1360780	−0.13	−0.49–0.22	.468	0.05	−0.38–0.49	.814	−0.02	−0.44–0.40	0.920
	Interaction	−2.37	−5.76–1.03	.172	0.12	−3.14–3.37	.945	−1.39	−5.28–2.51	0.485
Promoter	CpG 1	2.02	0.48–3.55	**.010**	−0.41	−2.01–1.19	.616	0.79	−1.10–2.67	0.413
	rs1360780	−0.06	−0.41–0.29	.728	−0.07	−0.45–0.31	.715	−0.04	−0.43–0.35	0.850
	Interaction	−0.63	−2.85–1.59	.578	0.88	−1.27–3.02	.423	0.15	−2.45–2.75	0.909
	CpG 2 (11)	0.48	−0.75–1.70	.446	−0.65	−1.97–0.68	.340	−0.39	−1.72–0.94	0.564
	rs1360780	−0.17	−0.51–0.17	.336	−0.13	−0.50–0.25	.510	−0.13	−0.52–0.25	0.495
	Interaction	0.69	−1.11–2.49	.452	1.27	−0.92–3.47	.255	2.03	0.06–4.02	**0.044**

N.B Values in bold are nominally significant. Highlighted values are significant after correction for multiple testing.

### 4.4 DNA methylation and gene expression

No correlation was found between changes in DNA methylation at any CpG site and changes in *FKBP5* expression (*p* >.05 for all probes, Supporting Information Table S3).

## 5 DISCUSSION

In this study we provide novel evidence that DNA methylation changes at *FKBP5* intron 7 are associated with response to exposure‐based cognitive behavior therapy in adults with anxiety disorders. In our sample, individuals who responded more positively to therapy had a reduction in DNA methylation at one CpG site of intron 7, while those who showed a poorer response had little change or an increase in DNA methylation. This effect remained significant after correcting for multiple testing. No significant interaction with rs1360780 genotype was detected for the CpG sites of intron 7. This finding is interesting as it has previously been demonstrated that a reduction in DNA methylation at this region is associated with higher levels of childhood trauma (albeit in an allele‐specific manner, Klengel et al., 2013). It has been suggested that *FKBP5* may represent a “differential susceptibility” gene (Vanzomeren‐Dohm, Pitula, Koss, Thomas, & Gunnar, 2015), conferring a higher sensitivity to the environment “for better or for worse”. Taken with the earlier findings with respect to child abuse, our results suggest that reduced DNA methylation at intron 7 may underlie a greater impact of both positive and negative environments. However, it should be noted that the aforementioned study of *FKBP5* intron 7 DNA methylation and childhood trauma found no association with recent life events (Klengel et al., 2013), suggesting that *FKBP5* demethylation could represent a stable epigenetic pattern, rather than reflecting rapid environmental changes. Nevertheless, in our sample, changes in DNA methylation during the course of therapy were observed for the majority of individuals, implying that dynamic changes are possible across the time period measured here.

We also observed a nominally significant effect on outcome of an interaction between rs1360780 and changes in percentage DNA methylation at the promoter region of *FKBP5*. At this CpG site, individuals with the risk genotype (CT/TT) showed an increase in DNA methylation associated with better treatment response, and a decrease in DNA methylation associated with poorer treatment response. In contrast, no association was seen in individuals homozygous for the C allele. It should be noted that this effect did not survive correction for multiple testing, likely a result of lack of power due to the relatively small sample size. Interestingly, we previously reported allele‐specific DNA methylation changes at the same CpG site (Roberts et al., 2015) were associated with response to CBT in children with anxiety disorders. However, in the earlier sample, we observed the opposite direction of effect, whereby better treatment response corresponded with an increase in DNA methylation, and poorer treatment response with decreases in DNA methylation in risk genotype individuals. These findings suggest that the T allele of rs1360780 may confer greater variability in biological changes, but the directionality is not yet clear. It is possible that the relatively small sample sizes also contribute to the observed imprecision. In addition, while the current study utilizes a similar design, there were a number of differences between the two samples. First, all participants in our previous report were children (mean age 9 years), while the current sample are adults. Second, all children received CBT, while the adults received exposure‐based therapies. Third, a wide range of anxiety diagnoses were included in the previous study, whereas the current study focuses primarily on agoraphobia and panic disorder, and specific phobias. These cohort and treatment differences may therefore also account for the lack of continuity between the results.

Intriguingly, the results of this study provide further evidence that changes at a biological level may be more important for longer term changes in symptomology. Change in DNA methylation from pre‐ to post‐treatment showed a strong association with treatment outcome at *follow‐up*, but no association with treatment outcome at posttreatment. This is in line with previous findings from our team, demonstrating DNA methylation changes during active treatment in both *FKBP5* and *SERT* genes that were associated with outcome at follow‐up (Roberts et al., 2015, 2014). Additionally, in this study we had data available for the follow‐up time‐point, but this did not show a significant association with treatment outcome at follow‐up. These findings suggest that epigenetic processes such as DNA methylation may potentially have a mechanistic role in the efficacy of psychological therapies. DNA methylation levels, particularly within promoter regions, are known to have an impact on subsequent gene expression. However, we found no correlation between observed changes in DNA methylation at any CpG site and *FKBP5* expression, and as such are not able to make any conclusions regarding the downstream effect of these processes in our sample––although it should be noted that DNA methylation and gene expression were determined from separate samples.

A number of caveats should be considered when interpreting the results of this study. First, while the current sample represents one of the largest reported for epigenetic studies of therapy response, it is still relatively small. Statistical power estimates (conducted using the powerreg package in Stata) suggested that the current sample had around 80% power to detect an effect of a similar magnitude to that detected for the CpG site in intron 7 with our corrected threshold of α = 0.0016, though considerably larger samples would be needed to accurately detect any smaller effects. As such, the study may be underpowered, particularly to identify significant interactions, and further research should aim to utilize larger samples where possible. However, the inclusion of a follow‐up time‐point is a particular strength of the study, and the use of a continuous outcome measure affords greater power. Second, in this study we have used clinician rated severity of the treated diagnosis as the primary treatment outcome. While the CGI is a well‐recognized and validated measure, it only concerns the severity of disorder. Another important aspect of treatment outcome in clinical psychology and psychiatry, particularly in fear‐related anxiety disorders such as phobias, is reduction in impairment. Further work in this area should consider changes in levels of impairment as well as diagnosis severity. However, we have focused on fear‐related anxiety diagnoses as exposure is a key element in their treatment, and it is therefore possible that any detectable association between epigenetic changes and phenotypic outcome will be seen at the level of symptoms, rather than acting in a disorder specific manner. Third, no information was available on early abuse or historical experiences of trauma in these participants. This is especially pertinent given the previous research identified early trauma‐related demethylation of *FKBP5*. Future studies including both historical data regarding life events and treatment outcomes may have the potential to fully investigate the potential association between *FKBP5* methylation and both positive and negative environmental influences. Fourth, the study sample included here contained some participants currently taking anxiolytic medications. While we included information regarding medication use at baseline in our statistical models, it is possible that our results may be confounded by the effect of medication use on DNA methylation. Finally, the repeated measures design in a clinical setting necessitates the use of peripheral samples, in this case whole blood DNA and RNA. Differences in the collection of such samples, such as time of day, food and drink intake, seasonality, and sample processing may influence the subsequent data derived from them. Importantly, tissue‐specific DNA methylation signatures have previously been identified (Davies et al., 2012), and as such we are unable make any conclusions about the relevance of these findings for any other tissues of interest, namely the brain. Furthermore, whole blood samples also consist of a heterogeneous cell mixture, and no information was available for the proportions of cell types within these samples. As DNA methylation may vary according to cell type, particularly in relation to the wide age range of our sample, this may represent an important potential confounder contributing to the findings of this study. However, for a potential biomarker of treatment outcome to have utility in clinical practice, it must be easily accessed from available sources, and thus the use of peripheral tissues represents a realistic approach for studies of this kind.

## 6 CONCLUSION

In this study, we demonstrate changes in DNA methylation at *FKBP5* intron 7 associated with reduction in anxiety severity following exposure‐based CBT. We also observed allele‐specific methylation at the promoter region associated with treatment response, although this did not reach a revised level of significance when correcting for multiple testing. Nevertheless, the results of this study add to the growing literature demonstrating the role of biological processes such as DNA methylation in response to environmental influences.

## CONFLICT OF INTEREST

Breen is a consultant in preclinical genetics for Eli Lilly. All other authors declared no financial interests.

## Supporting information

Appendix S1: Supplementary Materials
