# Supplementary material for: DNA methylation of FKBP5 and response to exposure‐based psychological therapy
Source: Am J Med Genet B Neuropsychiatr Genet. 2018 Oct 18;180(2):150–8. doi: 10.1002/ajmg.b.32650 (PMC6600698; doi:10.1002/ajmg.b.32650)
Supplement: Supplementary file 1 — Appendix S1: Supplementary Materials [file AJMG-180-150-s001.docx]

**Figure S1. Sample and treatment protocols**

**Table S1. Primer sequences**

| Amplicon | Length | Primer sequence (tags in lower case) | |
| --- | --- | --- | --- |
| *FKBP5* intron 7 | 497bp | F: | aggaagagag TTAAGGAGGTATGTTGTTTTTGGAA |
|  |  | R: | cagtaatacgactcactatagggagaaggctTCAAATTTATCTCTTACCTCCAACACTA |
| *FKBP5* promoter | 479bp | F: | aggaagagagTTGATTTAGTAGTTGGGTAAGTGGG |
|  |  | R: | cagtaatacgactcactatagggagaaggctACCCTATATCCCTCTTTTCTCCTAAA |

**Figure S2 – FKBP5 intron 7 sequence and probes**

Genomic location: chr6:35,558,298-35,558,794 (UCSC NCBI37/h19)


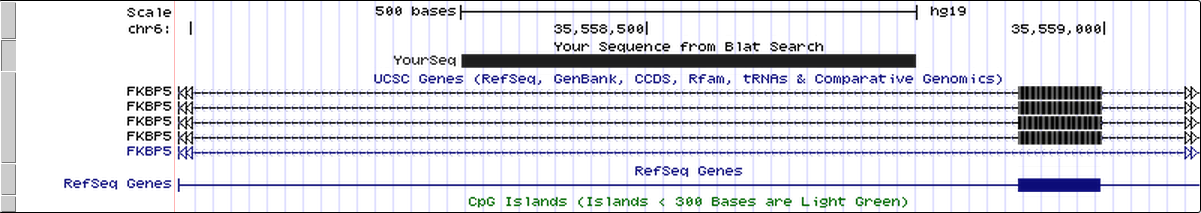


Amplicon sequence and probes

CpG 1

TCAAGGAGGCATGCTGTTTCTGGAATCCAAGGCAACTGACAAATTCTCTCTCTTCTCTACTTGGAGAAGTATAAAAAAAAAATGGCTTCGGGTTAGCTGCTTTCTTTCTTGTATC

CpG 2 CpG 3 CpG 4

TCTGGTCACAGAGCCTAGTGGCCCTCGAGGACTTGCAGTTGGGATAACAACTTGGAGCCACAGTGCAGGCCTCTTCGTGACTCCTGTGAAGGGTACAATCCGTTCAGCTCT

“Bin 2”

GAAAAGCTGCACCCCACTCCCCCAAGGAGCCACTTGGCAGAACGTGAACCTTTCTGTCCTCAACCCAGGAAAAAAAAAGTACAAAAAGAACAAGTCTAGGAACAAATAAGGG

CpG 5

AACAAGTCTTGGATTCTACCCAAAAAAGTTAAAAAAAAAAAAAAAAGCTGACACATAGGAACAAAATAAGAACACGGAGCTCCTTCGTTGTATATCAGCTGTGCTATGTCAGTTGTTCTATTCTTCAGCAGCAGTGTTGGAGGCAAGAGACAAACTTGA

**Figure S3 - *FKBP5* promoter sequence and probes**

Genomic location: chr6:35,695,823-35,696,542 (UCSC NCBI37/h19)


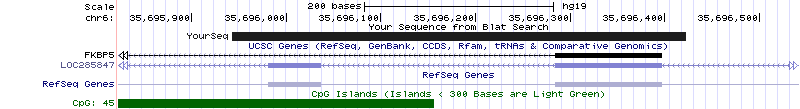


Amplicon sequence and probes

CpG 1

CCTGACTCAGCAGCTGGGTAAGTGGGTGTGCTCGCTCACCAGATCAACCGCTCTTGCAGCCTGCGGTCAGCGACGACCCGGCACTCGGCAGCTCCCTG

CpG 2

GAACGGGAGGGGCAAGCATACGGCCCGCTCCTCTGCTCGCGCCCCAGCCTGCCGCGCGAGACCTCCACGTGGCCGGTTTGCGCATCCCTCTGCCTTCTCGCCCCTCTATTAAAGCGTCTCCCAGCCCCCAGCCTGAGCGCAGCAGGCTCTGCCCACCTGGGAGAAGCACTCTCCTCACCCCACCCGACAGGTGTGGCCAGGCACACAGCTGCTGTGTACATGTCCTGTTTTCCCCAAATCTCACCCCTTTGGGACGCCTCGGAAGGTCTTGGCATGGGGGATGTAACTCTTAGTTGCAAACAGAGGTAGGAGTTTGTTCTGGGGTAGTGGTGGAGTAGGGAGCCGAGGAAGGGGATCCTAGGAGAAAAGAGGGACATAGGGC

**Table S2– Models testing the association between DNA methylation and treatment outcome**

|  |  |  | Post-treatment | | | | | | | |  | | |  | | | |  | | | |  |  |  |  |  |  |  |  |  |  |  |  |
| --- | --- | --- | --- | --- | --- | --- | --- | --- | --- | --- | --- | --- | --- | --- | --- | --- | --- | --- | --- | --- | --- | --- | --- | --- | --- | --- | --- | --- | --- | --- | --- | --- | --- |
| Region | | Test statistic | CpG Site | | Anxiety medication | | Time | | No. Sessions | |  | | | PC1 | | | |  | | | |  |  |  |  |  |  |  |  |  |  |  |  |
| FKBP5 | CpG 1 | β | 0.95 | | -1.76 | | 0 | | -0.01 | |  | | |  | | | |  | | | |  |  |  |  |  |  |  |  |  |  |  |  |
|  |  | CI (95%) | -1.01-2.92 | | -2.55--0.98 | | -0.00-0.00 | | -0.05-0.02 | |  | | | na | | | |  | | | |  |  |  |  |  |  |  |  |  |  |  |  |
|  |  | p | 0.342 | | <0.001 | | 0.812 | | 0.503 | |  | | |  | | | |  | | | |  |  |  |  |  |  |  |  |  |  |  |  |
|  | CpG 2 | β | 0.52 | | -1.74 | | 0 | | -0.01 | |  | | |  | | | |  | | | |  |  |  |  |  |  |  |  |  |  |  |  |
|  |  | CI (95%) | -1.02-2.06 | | -2.54--0.93 | | -0.00-0.00 | | -0.04-0.03 | |  | | | na | | | |  | | | |  |  |  |  |  |  |  |  |  |  |  |  |
|  |  | p | 0.510 | | <0.001 | | 0.726 | | 0.639 | |  | | |  | | | |  | | | |  |  |  |  |  |  |  |  |  |  |  |  |
|  | CpG 3 | β | -0.32 | | -1.7 | | 0 | | -0.01 | |  | | |  | | | |  | | | |  |  |  |  |  |  |  |  |  |  |  |  |
|  |  | CI (95%) | -1.08-0.45 | | -2.48--0.93 | | -0.00-0.00 | | -0.04-0.02 | |  | | | na | | | |  | | | |  |  |  |  |  |  |  |  |  |  |  |  |
|  |  | p | 0.415 | | <0.001 | | 0.941 | | 0.538 | |  | | |  | | | |  | | | |  |  |  |  |  |  |  |  |  |  |  |  |
|  | CpG 4 | β | 0.07 | | -1.74 | | 0 | | -0.01 | |  | | |  | | | |  | | | |  |  |  |  |  |  |  |  |  |  |  |  |
|  |  | CI (95%) | -2.11-2.25 | | -2.52--0.96 | | -0.00-0.00 | | -0.04-0.02 | |  | | | na | | | |  | | | |  |  |  |  |  |  |  |  |  |  |  |  |
|  |  | p | 0.951 | | <0.001 | | 0.806 | | 0.562 | |  | | |  | | | |  | | | |  |  |  |  |  |  |  |  |  |  |  |  |
|  | CpG 5 | β | -0.94 | | -1.7 | | 0 | | -0.02 | |  | | |  | | | |  | | | |  |  |  |  |  |  |  |  |  |  |  |  |
|  |  | CI (95%) | -2.34-0.46 | | -2.49--0.92 | | -0.00-0.00 | | -0.05-0.02 | |  | | | na | | | |  | | | |  |  |  |  |  |  |  |  |  |  |  |  |
|  |  | p | 0.189 | | <0.001 | | 0.968 | | 0.339 | |  | | |  | | | |  | | | |  |  |  |  |  |  |  |  |  |  |  |  |
|  | Bin 2 | β | -0.14 | | -1.78 | | 0 | | -0.02 | |  | | | -3.02 | | | |  | | | |  |  |  |  |  |  |  |  |  |  |  |  |
|  |  | CI (95%) | -1.50-1.79 | | -2.50--1.06 | | -0.00-0.00 | | -0.05-0.01 | |  | | | -6.67-0.64 | | | |  | | | |  |  |  |  |  |  |  |  |  |  |  |  |
|  |  | p | 0.864 | | <0.001 | | 0.453 | | 0.213 | |  | | | 0.106 | | | |  | | | |  |  |  |  |  |  |  |  |  |  |  |  |
| Promoter | CpG 1 | β | 1.74 | | -1.71 | | 0 | | 0 | |  | | |  | | | |  | | | |  |  |  |  |  |  |  |  |  |  |  |  |
|  |  | CI (95%) | 0.63-2.85 | | -2.37--1.05 | | -0.00-0.00 | | -0.03-0.03 | |  | | | na | | | |  | | | |  |  |  |  |  |  |  |  |  |  |  |  |
|  |  | p | **0.002** | | <0.001 | | 0.937 | | 0.938 | |  | | |  | | | |  | | | |  |  |  |  |  |  |  |  |  |  |  |  |
|  | CpG 2 | β | 0.85 | | -1.71 | | 0 | | 0 | |  | | |  | | | |  | | | |  |  |  |  |  |  |  |  |  |  |  |  |
|  |  | CI (95%) | -0.04-1.74 | | -2.34--1.07 | | -0.00-0.00 | | -0.03-0.03 | |  | | | na | | | |  | | | |  |  |  |  |  |  |  |  |  |  |  |  |
|  |  | p | 0.063 | | <0.001 | | 0.907 | | 0.786 | |  | | |  | | | |  | | | |  |  |  |  |  |  |  |  |  |  |  |  |
|  |  |  | Follow-up | | | | | | | |  | |  | | |  | | |  | | Change in DNA methylation at post-treatment and outcome at follow-up | | | | | |  | |  | |  | |  |
| Region | | Test statistic | CpG Site | Anxiety medication | | Time | | No. Sessions | | Age | | Smoking | | | Other medications | | PC1 | | | CpG Site | | | Anxiety medication | Time | No. Sessions | Age | | Smokinh | | Other medications | | PC1 | |
| FKBP5 | CpG 1 | β | -0.5 | -1.17 | | 0 | | -0.01 | |  | |  | | |  | |  | | | 0.52 | | | -1.18 | 0 | 0 |  | |  | |  | |  | |
|  |  | CI (95%) | -2.57-1.58 | -2.08--0.26 | | -0.00-0.00 | | -0.05-0.03 | | na | | na | | | na | | na | | | -1.70 -2.73 | | | -2.08--0.28 | -0.00-0.00 | -0.04-0.03 | na | | na | | na | | na | |
|  |  | p | 0.640 | 0.011 | | 0.744 | | 0.668 | |  | |  | | |  | |  | | | 0.648 | | | 0.010 | 0.275 | 0.911 |  | |  | |  | |  | |
|  | CpG 2 | β | 0.54 | -1.18 | | 0 | | -0.01 | |  | |  | | |  | |  | | | 1.03 | | | -1.2 | 0 | 0 |  | |  | |  | |  | |
|  |  | CI (95%) | -1.21-2.29 | -2.10--0.27 | | -0.00-0.00 | | -0.04-0.03 | | na | | na | | | na | | na | | | -0.56 -2.61 | | | -2.09--0.31 | -0.00-0.00 | -0.03-0.04 | na | | na | | na | | na | |
|  |  | p | 0.545 | 0.011 | | 0.774 | | 0.718 | |  | |  | | |  | |  | | | 0.205 | | | 0.008 | 0.259 | 0.864 |  | |  | |  | |  | |
|  | CpG 3 | β | -0.93 | -1.11 | | 0 | | 0 | | 0.02 | |  | | | -0.20 | |  | | | -0.85 | | | -1.09 | 0 | 0 | 0.01 | |  | | -0.26 | |  | |
|  |  | CI (95%) | -1.69- -0.17 | -1.99- -0.23 | | -0.00-0.00 | | -0.03-0.03 | | -0.00-0.04 | | na | | | -0.66-0.26 | | na | | | -1.63- -0.07 | | | -1.97--0.22 | -0.00-0.00 | -0.03-0.03 | -0.00-0.3 | | na | | -0.71-0.19 | | na | |
|  |  | p | **0.016** | 0.013 | | 0.428 | | 0.910 | | 0.071 | |  | | | 0.402 | |  | | | **0.032** | | | 0.014 | 0.373 | 0.813 | 0.131 | |  | | 0.258 | |  | |
|  | CpG 4 | β | -0.57 | -1.21 | | 0 | | -0.00 | | 0.02 | |  | | |  | |  | | | 1.09 | | | -1.24 | 0 | 0 | 0.01 | |  | |  | |  | |
|  |  | CI (95%) | -1.87-0.72 | -2.11--0.33 | | -0.00-0.00 | | -0.04-0.03 | | -0.00-0.04 | | na | | | na | | na | | | -1.42-3.61 | | | -2.12--0.35 | -0.00-0.00 | -0.03-0.04 | -0.01-0.03 | | na | | na | | na | |
|  |  | p | 0.387 | 0.007 | | 0.581 | | 0.797 | | 0.126 | |  | | |  | |  | | | 0.396 | | | 0.006 | 0.327 | 0.872 | 0.166 | |  | |  | |  | |
|  | CpG 5 | β | -1.65 | -1.2 | | 0 | | -0.01 | |  | |  | | |  | |  | | | -2.51 | | | -1.11 | 0 | -0.01 |  | |  | |  | |  | |
|  |  | CI (95%) | -3.09- -0.22 | -2.06--0.33 | | -0.00-0.00 | | -0.04-0.03 | | na | | na | | | na | | na | | | -3.90 - -1.13 | | | -1.95--0.28 | -0.00-0.00 | -0.04-0.02 | na | | na | | na | | na | |
|  |  | p | **0.024** | 0.007 | | 0.876 | | 0.706 | |  | |  | | |  | |  | | | **3.90E-04** | | | 0.009 | 0.653 | 0.550 |  | |  | |  | |  | |
|  | Bin 2 | β | -1.30 | -1.13 | | 0 | | -0.02 | | 0.017 | |  | | | -0.34 | | -0.96 | | | -1.26 | | | -1.09 | 0 | -0.02 | 0.01 | |  | | -0.42 | | -0.67 | |
|  |  | CI (95%) | -2.73-0.12 | -2.03--0.25 | | -0.00-0.00 | | -0.05-0.02 | | -0.00-0.04 | | na | | | -0.82-0.15 | | -5.04-3.12 | | | -3.15 -0.63 | | | -1.97- -0.21 | -0.00-0.00 | -0.05-0.02 | -0.01-0.03 | | na | | -0.89-0.06 | | -4.84-3.51 | |
|  |  | p | 0.072 | 0.012 | | 0.824 | | 0.316 | | 0.099 | |  | | | 0.172 | | 0.645 | | | 0.192 | | | 0.016 | 0.985 | 0.346 | 0.197 | |  | | 0.084 | | 0.753 | |
| Promoter | CpG 1 | β | 0.08 | -0.95 | | 0 | | 0.01 | |  | |  | | |  | |  | | | 0.87 | | | -1.42 | 0 | 0 |  | |  | |  | |  | |
|  |  | CI (95%) | -1.01-1.16 | -1.75--0.15 | | -0.00-0.00 | | -0.02-0.04 | | na | | na | | | na | | na | | | -0.44 -2.17 | | | -2.17--0.67 | -0.00-0.00 | -0.03-0.03 | na | | na | | na | | na | |
|  |  | p | 0.889 | 0.02 | | 0.135 | | 0.599 | |  | |  | | |  | |  | | | 0.192 | | | <0.001 | 0.442 | 0.823 |  | |  | |  | |  | |
|  | CpG 2 | β | -0.16 | -0.97 | | 0 | | 0.01 | |  | | -0.11 | | |  | |  | | | 0.55 | | | -1.43 | 0 | 0 |  | | -0.01 | |  | |  | |
|  |  | CI (95%) | -1.22-0.90 | -1.75--0.19 | | -0.00-0.00 | | -0.02-0.03 | | na | | -0.51-0.28 | | | na | | na | | | -0.45 -1.55 | | | -2.16--0.69 | -0.00-0.00 | -0.03-0.03 | na | | -0.41-0.38 | | na | | na | |
|  |  | p | 0.765 | 0.015 | | 0.161 | | 0.716 | |  | | 0.581 | | |  | |  | | | 0.281 | | | <0.001 | 0.478 | 0.861 |  | | 0.954 | |  | |  | |

**Table S3 – Changes in DNA methylation and changes in gene expression**

|  |  |  |  | Change in FKBP5 expression | |
| --- | --- | --- | --- | --- | --- |
|  |  |  |  | Pre- to Post-treatment | Pre-treatment to follow-up |
| Pre- to Post-treatment | Intron 7 | CpG 1 | corr | 0.0308 | -0.0635 |
|  |  |  | p | 0.7990 | 0.6043 |
|  |  | CpG 2 | corr | 0.1366 | 0.1142 |
|  |  |  | p | 0.2493 | 0.3430 |
|  |  | CpG 3 | corr | 0.0487 | 0.0141 |
|  |  |  | p | 0.6598 | 0.9002 |
|  |  | CpG 4 | corr | 0.0034 | -0.0097 |
|  |  |  | p | 0.9763 | 0.9334 |
|  |  | CpG 5 | corr | 0.1385 | -0.0657 |
|  |  |  | p | 0.2392 | 0.5833 |
|  | Promoter | CpG 1 | corr | 0.1248 | 0.0017 |
|  |  |  | p | 0.2700 | 0.9880 |
|  |  | CpG 2 | corr | 0.1037 | -0.0610 |
|  |  |  | p | 0.3662 | 0.6029 |
| Pre-treatment to follow-up | Intron 7 | CpG 1 | corr | 0.0137 | -0.1032 |
|  |  |  | p | 0.9106 | 0.4023 |
|  |  | CpG 2 | corr | 0.0194 | -0.1371 |
|  |  |  | p | 0.8741 | 0.2685 |
|  |  | CpG 3 | corr | -0.1521 | 0.0467 |
|  |  |  | p | 0.1698 | 0.6809 |
|  |  | CpG 4 | corr | 0.0360 | -0.0109 |
|  |  |  | p | 0.7526 | 0.9251 |
|  |  | CpG 5 | corr | -0.1612 | -0.0952 |
|  |  |  | p | 0.1701 | 0.4298 |
|  | Promoter | CpG 1 | corr | -0.0356 | 0.0813 |
|  |  |  | p | 0.7551 | 0.4851 |
|  |  | CpG 2 | corr | -0.1720 | -0.0061 |
|  |  |  | p | 0.1321 | 0.9583 |
